# Effect of mesenchymal stem cells transplantation on the changes of oligodendrocyte lineage in rat brain with experimental autoimmune encephalomyelitis

**DOI:** 10.1002/brb3.1999

**Published:** 2020-12-14

**Authors:** Jun‐Mei Zhang, Hua Wang, Yu‐Ying Fan, Feng‐Hua Yang

**Affiliations:** ^1^ Department of Pediatrics Shengjing Hospital of China Medical University Shenyang China

**Keywords:** bone marrow mesenchymal stem cells, experimental autoimmune encephalomyelitis, oligodendrocyte lineage, transplantation

## Abstract

**Objective:**

To explore the effect of bone marrow mesenchymal stem cells (BM‐MSCs) transplantation on the changes of oligodendrocyte lineage in brain of experimental autoimmune encephalomyelitis (EAE) rats.

**Methods:**

The animals were divided into normal control group, EAE model group (EAE group), cell culture medium injection treatment group (placebo treatment group), and MSCs treatment group (treatment group). The changes of A2B5‐, O4‐, and CNPase‐positive cells in oligodendrocyte lineage in rat brain were observed after 1, 3, 7, 14, 21, and 28 days.

**Results:**

The number of A2B5‐positive cells in rat brain of the treatment group at each time point was significantly more than that of the EAE and placebo treatment groups, and most obvious at 14 days. The O4‐positive cells number at each time point in the treatment group was significantly increased compared with the EAE and placebo treatment groups, and most obvious at 14 days. The CNPase‐positive cells number at each time point in the treatment group was significantly increased compared with the EAE and placebo treatment groups, and most obvious at 14 days.

**Conclusions:**

MSCs treatment can increase cells expression in oligodendrocyte lineage, which laying a solid foundation for myelin regeneration.

## Graphical abstract

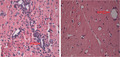

## 1 INTRODUCTION

Acute disseminated encephalomyelitis (ADEM) is common childhood central nervous system demyelinating disease, cellular and humoral immunity participate in the pathogenesis and lead to brain and spinal cord white matter damage‐based diseases. At present, the exact etiology and pathogenesis are not yet fully understood, and easy to leave a permanent neurological sequelae. In ADEM lesions, the involved cells are mainly oligodendrocytes (OLG), showing varying degrees of loss.

The main function of OLG is the formation of nerve myelin sheath, so the repair of myelin sheath is fundamentally the repair of OLG. How to minimize the damage of OLG, and how to promote the differentiation of endogenous glial precursor cells into functional OLG, as well as how to promote the repair of central nervous system injury through exogenous cell transplantation, has been one of the hot spots of domestic and foreign scholars’ study.

Experimental autoimmune encephalomyelitis (EAE) has the same clinical, biochemical, immunological, and pathological features as acute disseminated encephalomyelitis (ADEM), and is a classical animal model of ADEM (El Behi et al, 2005).

The pathogenesis of EAE mainly includes (a) Primary demyelination, at this time few oligodendrocyte damage could be observed at this time; (b) Widely lost of OLG during demyelination process; (c) Primary oligodendrocyte injury and lead to secondary demyelination; (d) A large number of macrophages are activated, and causing nonselective tissue damage, it cannot only involving the myelin sheath, but also involves axons and astrocytes cell. The damage and loss of OLG are the key link of EAE. Therefore, OLG play an important role in the damage and repair of EAE. In this study, EAE animal model was used and treated with MSCs intervention to observe the changes of oligodendrocyte lineage in EAE rats, and further clarify the repair process of damaged myelin sheath.

## 2 EXPERIMENTAL MATERIALS AND METHODS

### 2.1 Main instruments and reagents

#### 2.1.1 Instruments

Frozen microtome, fluorescence microscope, image acquisition, and analysis system.

#### 2.1.2 Reagents

A2B5 monoclonal antibody, mouse anti‐rat IGM‐type monoclonal antibody (abcam Company, US). O4 monoclonal antibody, mouse anti‐rat IGM‐type monoclonal antibody (abcom Company, US). CNPase monoclonal antibody, mouse anti‐rat IGG‐type monoclonal antibody (abcom Company, US). Goat anti‐mouse IGG+IGM+IGA‐FITC secondary antibody (CHEMICON Company, US), goat anti‐mouse IGG‐FITC secondary antibody, purchased from the Sino‐Forest Biological Products Co.

### 2.2 Preparation of EAE animal model and the neurological function score

#### 2.2.1 Preparation of EAE animal model

##### Experimental animals

Guinea pigs, 180–250 g, male and female; Wistar rats, female, 160–180 g, provided by Experimental Animal Center, Shengjing Hospital of China Medical University.

##### Preparation of complete antigen

Guinea pigs were selected and weighted, 5% chloral hydrate 6 ml/kg was used for abdominal injection anesthesia, the head, neck, and back hair was cut, and disinfection with iodine, then cut the skin from the middle, stripping the head, neck, back skin and muscle, and cut the occipital bone and spinal canal, and then quickly removed the spinal cord on the ice plate, after that, carefully peel off the spine, removed the cauda equina, and placed in the centrifuge tube and weighted, then the spinal cord was homogenize with saline at the ratio of 1:1 in ice‐bath to make guinea pig spinal cord homogenate (GPSCH), then fully mixed the homogenate and the equal volume of complete Freund's adjuvant (CFA), repeatedly beaten with sterile syringe to make water‐in‐oil emulsion, stored at 4°C, and used within 12 hr.

##### Preparation of EAE model

Wistar rats were weighed, labeled, and abdominal injection anesthetized with 5% chloral hydrate 6 ml/kg, then rejection with bordetella pertussis vaccine (BPV) stock solution 0.1 ml (containing 2 × 10^10^ bacteria) on the left back foot under sterile state, and injected the mixed solution of CFA and GPSCH 0.4 ml on the four foot pad.

##### Neurological function score of EAE model

0 point: No disease; 1 point: Tail tension was reduced or mild gait clumsy, double hind legs were slightly drag; 2 points: Tail had no tension or moderate gait clumsy, double hind legs were moderate weakness, obviously drag was observed or lack of the maintenance of posture; 3 points: Double hind limb was severe weakness, but still able to be dragged, and the limb was weakness; 4 points: Double hind limbs or limbs paralysis and incontinence; 5 points: Dying state.

The day of immunization antigen was 0 day, and after immunization, the rats eat and drank freely, and all rats were weighted every day and received functional score, the score of 2 points or more was considered as disease rats.

### 2.3 Culture and identification of BM‐MSCs

#### 2.3.1 Culture of BM‐MSCs

The male Wistar rats weighted 100 g were anesthetized, and the femur was removed under sterile conditions, removed the tissues attached to the surface of the bone. The bone marrow was rinsed with DMEM‐F12 medium containing 10% fetal bovine serum by a syringe to obtain cell suspension. The cell suspension was inoculated into a culture flask and cultured in a 37°C, 5% CO_2_ incubator. Discarded non‐adherent cells within 24 hours, and replaced the culture medium every 2–3 days, and then discarded the suspended cells. Passaged when the cells reached 80% fusion, and the cells that passaged three times were digested with 0.25% trypsin, and then identified by flow cytometry.

#### 2.3.2 Identification of BM‐MSCs

Selected the third generation of BM‐MSCs and digested with trypsin, and then centrifuged (4°C, 900 rpm, 6 min), discarded the supernatant, PBS resuspended and then centrifuged again, PBS resuspension (3 ml) with 5% fetal bovine serum, averagely divided into three parts and added into the centrifuge tube and marked (negative control, CD34, and CD90), then centrifuged, discarded the supernatant, added 200 µl PBS for resuspension, then added 2 µl fluorescent‐labeled goat anti‐rat CD34 and CD90IgG antibody according to the marker, drak cultured in the incubator at 37°C for 30 min, mixed with 5% serum PBS and centrifuged, discarded the supernatant, and then added 500 µl 5% serum PBS, mixed and put on the test machine. The results showed CD34 was negative and CD90 was positive, which confirmed as BM‐MSCs.

### 2.4 Animal grouping

All animals were divided into four groups: normal control group, EAE model group (EAE group), injection cell culture medium treatment group (placebo injection treatment group), and MSCs treatment group (treatment group). The specific method was as follow: A total of 218 Wistar rats were included, there were eight rats in the normal control group, and the rest 210 rats were given immune antigen to make EAE animal models, after modeling success, randomly selected 144 EAE rats with the functional score of 2 or more, and divided into EAE group, placebo injection treatment group and MSCs treatment group, 48 rats in each group; the three groups were, respectively, divided into six subgroups: 1, 3, 7, 14, 21, and 28 days post‐onset groups, eight rats in each group.

### 2.5 BM‐MSCs transplantation

The second or third generation of BM‐MSCs was used, discarded the culture medium and washed with PBS, then added trypsin for digestion, when the pseudo‐foot of cells completely shrink, added culture medium to stop, blow and beat even and then centrifuged (4°C, 800 rpm, 5 min), discarded the supernatant, and added 20 µl culture medium to resuspended, took 1 1 µl and added 100 µl culture medium for counting (required the number of cells reached 10^6^). All the applicated cells were put into the icebox. The rats received abdominal injection anesthesia with 5% chloral hydrate (6 ml/kg), fixed the limbs, and then disinfected the skin and hair on the head, then cut the head hair, and cut the skin in the middle with the disinfected knife, cotton swab to stop bleeding, fully exposed the anterior fontanel and burned the periosteum on the anterior fontanel with 3% hydrogen peroxide, then positioned with stereotaxic device (at the site 1.0 mm back of anterior fontanel and aside 1.5 mm, and at the depth of 3.5 mm), the bilateral ventricle was injected with prepared cells 10 µl, after injection, keep the needle inside for 2 min, and then withdraw the needle slowly, sutured the head skin, and put back to the cage and fed.

### 2.6 Materials

Abdominal injection anesthesia with 5% chloral hydrate (6 ml/kg), open the chest, inserted the trocar into the ascending aorta through the left ventricle, and cut the auricula dextra, pull of the trocar core and rapidly infused 200 ml 0.9% saline until the effluent becomes clear, then infused with 200 ml 4% paraformaldehyde from fast to slow till the lamb and tail stiffness, completed within 30 min. Took brain after fixed 1 hr, the whole brain tissue were fixed in 4% paraformaldehyde for 24 hr and then fixed in 20% sucrose solution for 24 hr, after the tissues sink to the bottom, selected the anterior fontanel and the posterior brain tissues, embedded with OCT, and cryostat microtome was used to conduct continuous coronal slice, the thickness of the slice was 10 µm, and then mounted on the polyglycine‐treated clean slides, stored at −80°C for immunohistochemistry.

### 2.7 Immunohistochemical fluorescence detection

(a) Took the slices stored at −80°C, buffered at −20°C for 30 min, then drying at room temperature. (b) Secondary fixation for 30 min with methanol. (c) Rinsed with PBS 3 × 5 min. (d) Antigen repair (cooled to room temperature after heat repair). (e) Rinsed with PBS 2 × 5 min. (f) Closed with 10% serum for 10 min. (g) Added A2B5 monoclonal antibody (10 µg/ml), O4 monoclonal antibody (10 µg/ml), and CNPase monoclonal antibody (10 µg/ml) on the tissue sections, then placed the slices into a wet box with a small amount of PBS, 4°C overnight. (h) Rinsed with PBS 3 × 5 min. (i) Added goat anti‐mouse IgG+IgM+IGA‐FITC (50 µg/ml) and secondary antibody goat anti‐mouse IgG‐FITC (100 µg/ml) 50 µl on the tissue sections, incubated in the 37°C wet box for 1.5 hr. Operated avoid the light from this step. (j) Rinsed with PBS 3 × 5 min. (k) Dry avoid the light, and observed under the fluorescent microscope (blue excitation light).

### 2.8 Observation on the positive cells under fluorescent microscope and counted

The morphology of A2B5‐, O4‐, and CNPase‐positive cells was observed under fluorescence microscope. Three consecutive sections were selected from each rat. Five 200‐fold visual fields were randomly selected in the white matter region. The number of A2B5‐, O4‐, and CNPase‐positive cells was counted, and the arithmetic mean of the positive cells of each rat was calculated, and expressed with number/fold.

### 2.9 Statistical analysis

All the data were expressed as *x* ± *s*, one‐way analysis of variance was used to analyze each time point in the experimental group, and *t* test was used for the comparison between two groups, *p* < .05 was considered significant, and *p* < .01 was considered highly significant.

### 2.10 Ethics approval

This study has been approved by the ethics committee of Shengjing Hospital of China Medical University.

## 3 RESULTS

### 3.1 Effect of MSCs transplantation on the clinical symptoms and clinical scores

**FIGURE 1:**
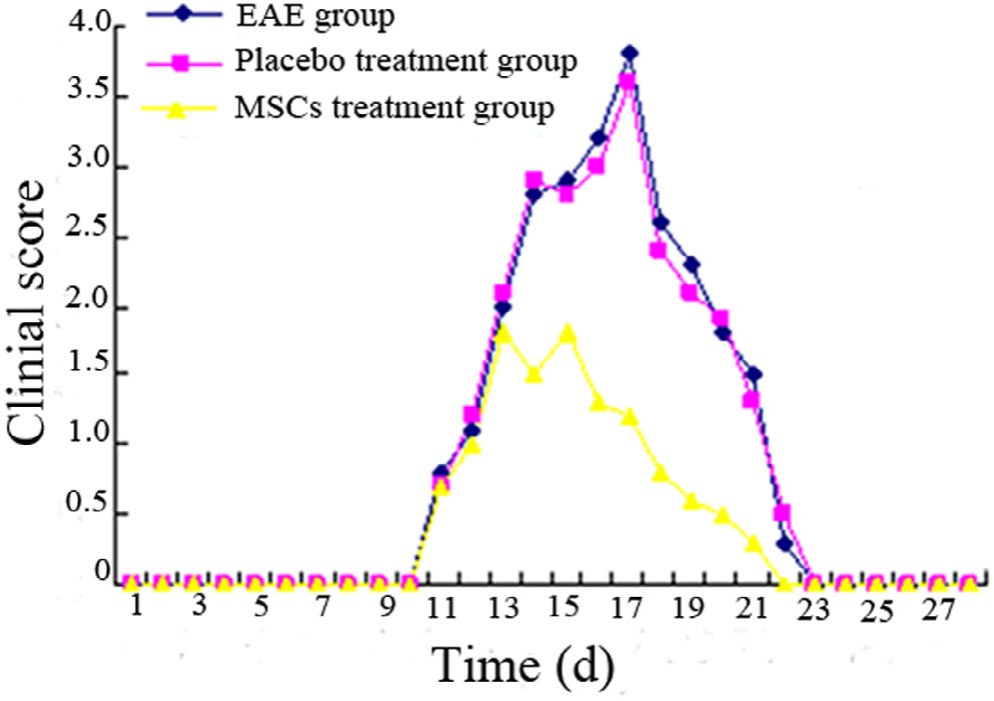
The changes of clinical scores of EAE group, placebo treatment group, and MSCs treatment group

The clinical symptoms of EAE group, placebo treatment group, and MSCs treatment group were compared to analyze the effect of MSCs transplantation on clinical symptoms in onset rats. The duration of the disease in the EAE group was as long as 12.13 ± 1.25 days; the duration of the placebo treatment group was 11.96 ± 1.32 days, which was not significantly different from that in the EAE group (*p* > .05). The duration of the disease in the MSCs treatment group was 4.32 ± 0.63, which was significantly different from the EAE group and the placebo treatment group (*p* < .05). In the functional scoring experiment, the highest average clinical score of the EAE group was 3.87 ± 0.23, and the highest clinical score of the placebo treatment group was 3.62 ± 0.28, which was not significantly different from the EAE group (*p* > .05). The highest clinical score of the MSCs treatment group was 2.35 ± 0.18, and there was significant difference between the EAE group and the MSCs treatment group (*p* < .05, Table 1). The changes of the clinical scores of the EAE group, the placebo treatment group, and the MSCs treatment group are shown in Figure 1.

**TABLE 1:** The changes of clinical symptoms of EAE group, placebo treatment group, and MSCs treatment group

Groups	*n*	Duration of clinical symptoms	Highest average clinical score
EAE	8	12.13 ± 1.25	3.87 ± 0.23
MSCs treatment	8	11.96 ± 1.32^a^	3.62 ± 0.28^a^
Placebo treatment	8	4.32 ± 0.63^b^, ^c^	2.35 ± 0.18^b^, ^c^

^a^ *p* > .05, compared between placebo treatment group and EAE group.

^b^ *p* < .05, compared between MSCs treatment group and EAE group.

^c^ *p* < .05, compared between MSCs treatment group and placebo treatment group.

### 3.2 Changes in HE staining in rat brain before and after transplantation

**FIGURE 2:**
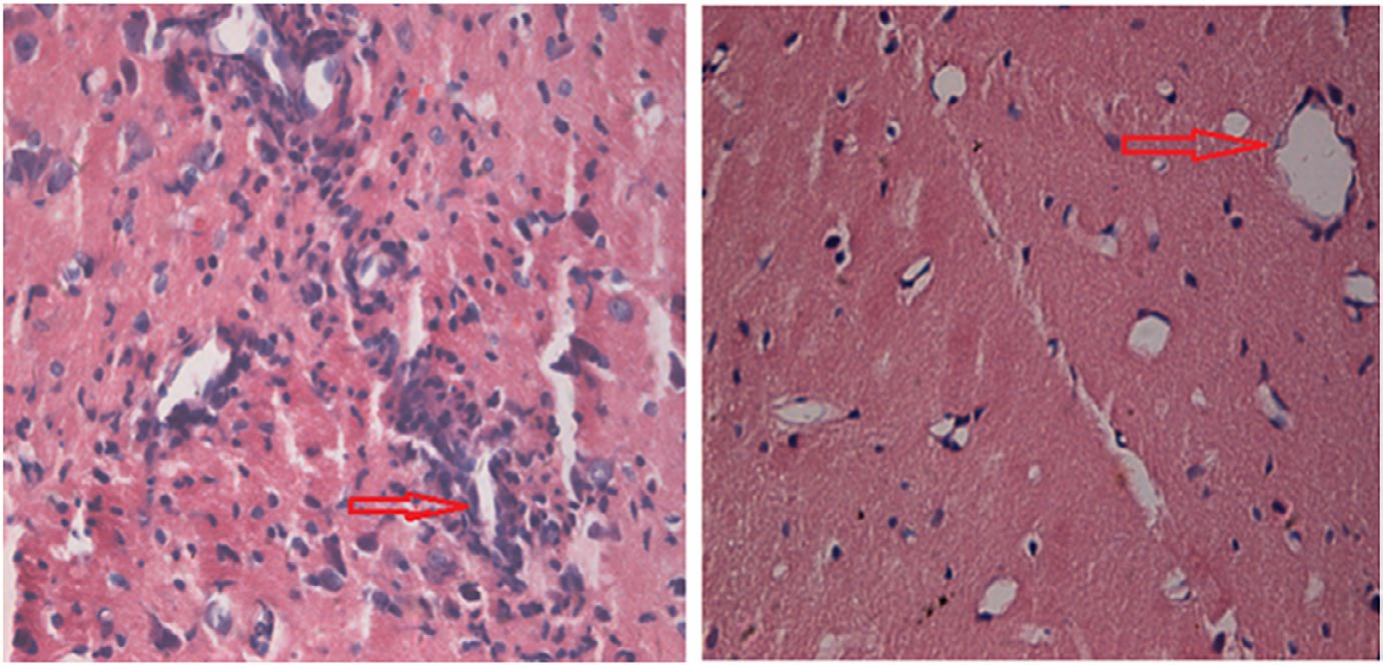
Changes in HE staining in rat brain before and after transplantation

In the EAE group, significant inflammatory cell infiltration around the blood vessels was observed, and a “vascular sheath”‐like change was formed around the blood vessels. This inflammatory infiltration was significantly improved on the third day after transplantation of MSCs (Figure 2).

### 3.3 Effect of MSCs transplantation on myelin structure changes

**FIGURE 3:**
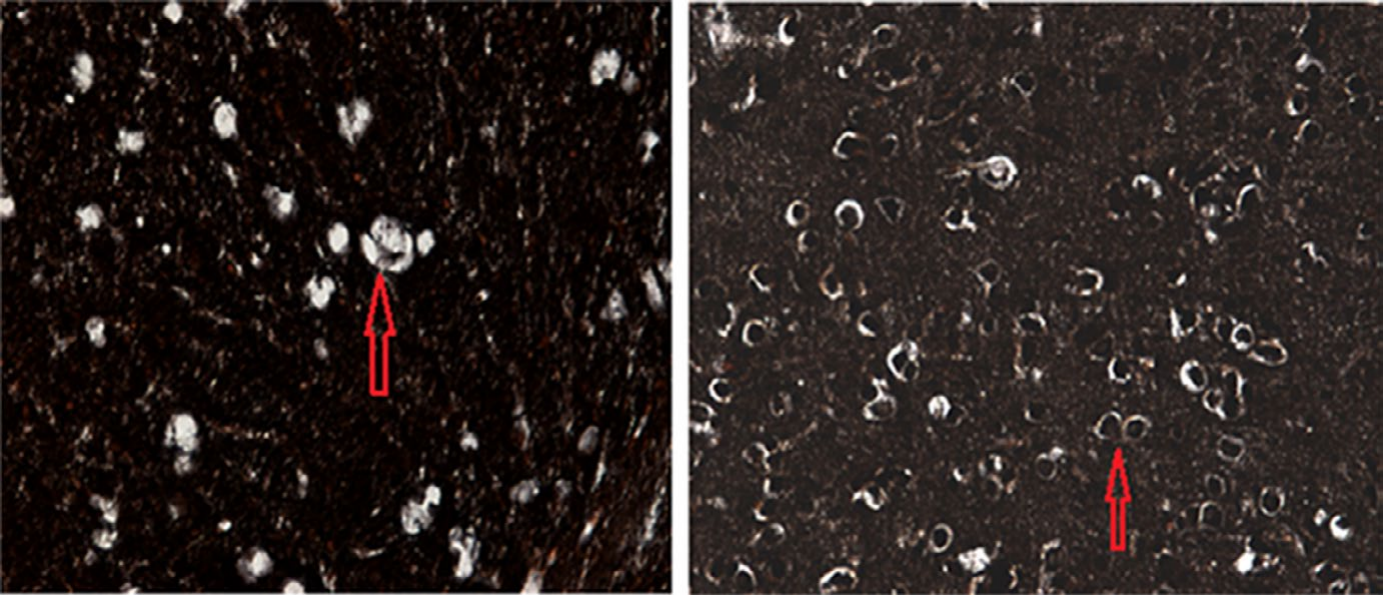
Effect of MSCs transplantation on myelin structure changes

In the control group, the myelin sheath samples were arranged neatly, without edema, stratification, fragmentation, and vacuolation formation. At 1 day after EAE treatment, the myelin sheath showed edema and partial rupture, fragmentation, disintegration, and aggregation. The myelin lesions were obvious at 3–7 days after EAE treatment, which showed as the expansion of myelin disintegration, diffuse distribution of myelin lesions, and obvious disorder of tissue structure. Part of the myelin sheath was vacuolated, and the number of myelin fragments was relatively reduced after 14 days. The myelin changes in the placebo treatment group were the same as those in the EAE group. The myelin sheath also showed extensive destruction at 1 day in the MSCs treatment group, and recovered gradually from 3 day, and the myelin recovery at different time points was better than that in the EAE group and the placebo treatment group (Figure 3).

### 3.4 Morphology and distribution of A2B5‐positive cells in normal rat brain

A2B5‐positive cells could be observed in normal adult rat brain, and most in the cortical white matter and cortical region, and the A2B5‐positive cells could also be observed in hippocampus, striatum, and cerebellum. A2B5 expression could be observed on the cell membrane of oligodendrocyte progenitor cells, and the A2B5‐positive cells showed yellow‐green color under fluorescence microscope, and the showed small form, rounded, oval, or fusiform shape.

### 3.5 Morphology and distribution of A2B5‐positive cells in rat brain of each group

**FIGURE 4:**
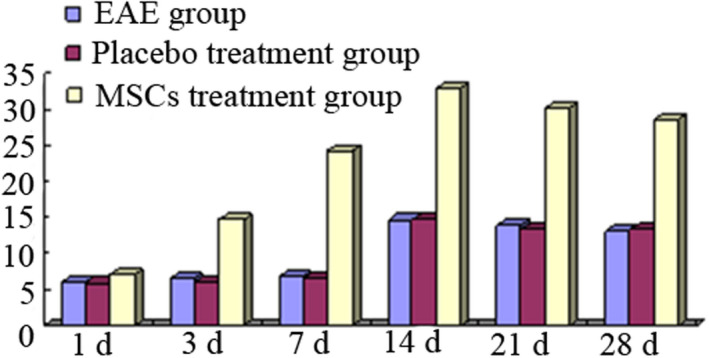
Changes of A2B5‐positive cells in rat brain of each group

In the 200‐fold microscope, the mean number of A2B5‐positive cells per field of cerebral cortex in each group was shown in Table 2. Less A2B5‐positive cells distributed in rat cerebral cortex in EAE 1 day group; the A2B5‐positive cells began to increase in EAE 3 days group and EAE 7 days group, and there was no significant difference compared with EAE 1 day group (*p* > .05); A2B5‐positive cells were most when EAE for 14 days, there was significant difference compared with EAE 1 day group (*p* < .05), and the A2B5‐positive cells were decreased again at 21 and 28 days. The change trend at each time point in placebo treatment group was same with EAE group, and there was no significant difference between two groups (*p* > .05). In the MSCs treatment group, the A2B5 cells were increased not obviously at 1 day, and increased significantly at 3 days, and reached peak from 7 to 14 days, then decreased, and there was significant difference in A2B5 cells compared with the EAE group and the placebo treatment group (*p* < .05) (Table 2, Figure 4).

**TABLE 2:** Changes of A2B5‐positive cells in rat brain (*n*/field, $\bar{X}$ ± *s*)

Groups	1 day	3 days	7 days	14 days	21 days	28 days
EAE	6.0 ± 4.0	6.6 ± 2.8	6.8 ± 3.8	14.5 ± 3.2^a^	13.8 ± 3.3^a^	13.1 ± 2.8^a^
Placebo treatment	5.8 ± 4.6	5.9 ± 3.8	6.5 ± 3.4	14.6 ± 4.2^a^	13.2 ± 3.4^a^	13.3 ± 2.6^a^
MSCs treatment	7.0 ± 3.2	14.6 ± 4.8^b^, ^c^	24.2 ± 3.4^b^, ^c^	32.8 ± 4.8^b^, ^c^	30.2 ± 4.6^b^, ^c^	28.4 ± 4.8^b^, ^c^

^a^ *p* < .05, compared with 1 day in the same group.

^b^ *p* < .05, compared between MSCs treatment group and EAE group.

^c^ *p* < .05, compared between MSCs treatment group and placebo treatment group.

### 3.6 Morphology and distribution of O4‐positive cells in normal rat brain

Same as the A2B5‐positive cells, there are more O4‐positive cells in the normal adult rats, and most in the cortical and subcortical white matter regions, O4‐positive cells could also be found in the cerebellum. The O4‐positive cells in the hippocampus region were distributed on both sides of the neuronal aggregation. O4 was expressed on the cell membrane of immature OLG and showed yellowish green under fluorescence microscope. O4‐positive cells were slightly larger than A2B5‐positive cells, the morphology was round or oval, and most of the O4‐positive cells were round.

### 3.7 Changes of number of O4‐positive cells in brain of rats in each group

**FIGURE 5:**
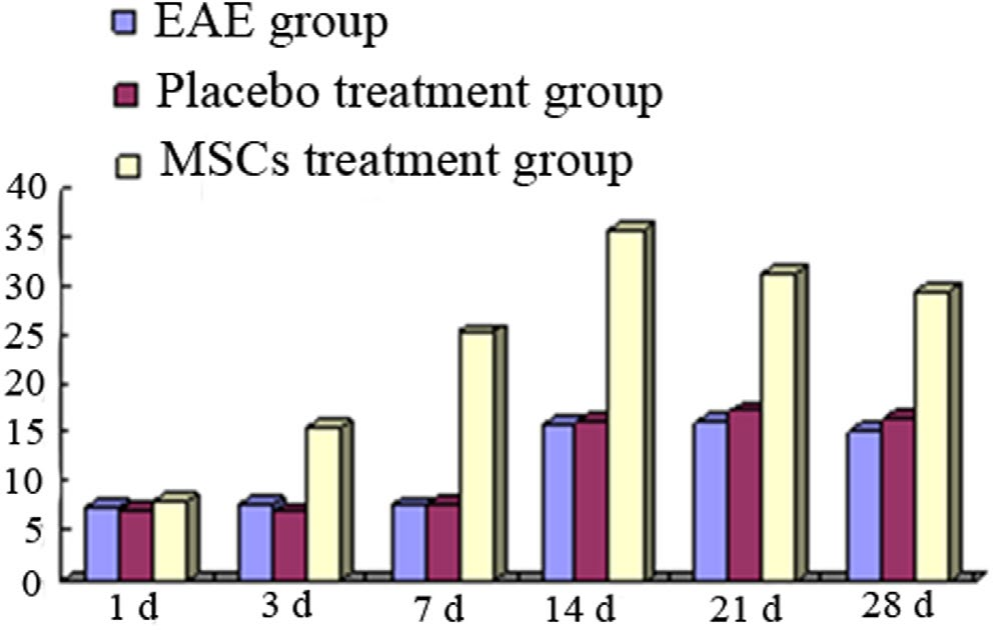
Changes of O4‐positive cells in rat brain in each gorup

In the 200‐fold microscope, the average number of O4‐positive cells per field of view in the cerebral cortex regions of each group is shown in Table 2. There were fewer O4‐positive cells in rat cerebral cortex in EAE 1 day group. O4‐positive cells began to increase in 3 days group and 7 days group, and there was no significant difference compared with that in 1 day group (*p* > .05), and the O4‐positive cells were the most in EAE 14 days, and there was significant difference in number of O4‐positive cells compared with EAE 1 day group (*p* < .05), the O4‐positive cells were decreased in 21 and 28 days group; the change trend at each time point in placebo treatment group was same with EAE group, and there was no significant difference between two groups (*p* > .05). In the MSCs treatment group, the O4‐positive cells were increased not obviously at 1 day, and increased significantly at 3 days, and reached peak from 7 to 14 days, then decreased, and there was significant difference in O4‐positive cells compared with the EAE group and the placebo treatment group (*p* < .05) (Table 3, Figure 5).

**TABLE 3:** Changes of O4‐positive cells in rat brain in each gorup (*n*/field, $\bar{X}$ ± *s*)

Groups	1 day	3 days	7 days	14 days	21 days	28 days
EAE	7.3 ± 4.2	7.6 ± 2.3	7.5 ± 3.1	15.8 ± 3.9^a^	16.2 ± 3.2^a^	15.2 ± 2.3^a^
Placebo treatment	7.1 ± 3.8	6.9 ± 4.6	7.8 ± 3.4	16.1 ± 3.6^a^	17.2 ± 3.6^a^	16.4 ± 3.3^a^
MSCs treatment	8.1 ± 3.8	15.6 ± 4.2^b^, ^c^	25.3 ± 4.3^b^, ^c^	35.7 ± 3.8^b^, ^c^	31.5 ± 4.2^b^, ^c^	29.4 ± 4.3^b^, ^c^

^a^ *p* < .05, compared with 1 day in the same group.

^b^ *p* < .05, compared between MSCs treatment group and EAE group.

^c^ *p* < .05, compared between MSCs treatment group and placebo treatment group.

### 3.8 Morphology and distribution of CNPase‐positive cells in normal rat brain

There were certain number of CNPase‐positive cells in normal adult rat brain, and the distribution was similar with A2B5‐ and O4‐positive cells, which most in the cortical and subcortical white matter area. CNPase‐positive cells in the cortical area were scattered, and most were distributed on the area above and below the internal pyramidal layer. CNPase‐positive cells in the hippocampus also distributed on both sides of neuronal aggregation. CNPase‐positive cells in the white matter area were evenly distributed. CNPase were expressed in mature OLG loose myelin cytoplasm, and the volume of CNPase‐positive cells was slightly larger than A2B5‐ and O4‐positive cells, which showed round or oval shape, and most cells were round, and there were more protrusions.

### 3.9 Changes of number of CNPase‐positive cells in brain of rats in each group

**FIGURE 6:**
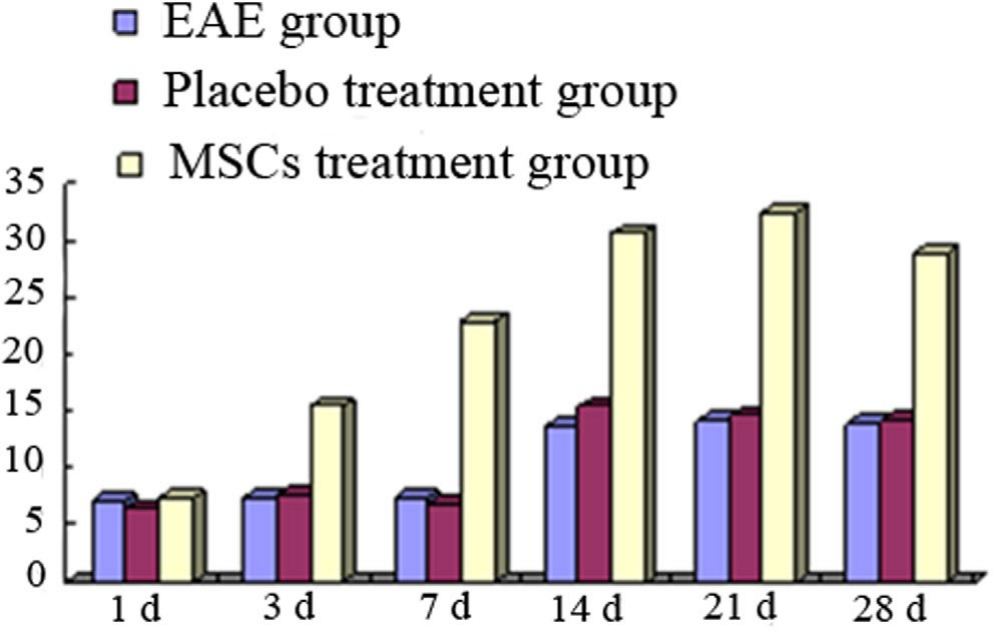
Changes of CNPase‐positive cells in rat brain in each group

In the 200‐fold microscope, the average number of CNPase‐positive cells per field of view in the cerebral cortex regions of each group is shown in Table 2. There were fewer CNPase‐positive cells in rat cerebral cortex in EAE 1 day group. CNPase‐positive cells began to increase in 3 days group and 7 days group, and there was no significant difference compared with that in 1 day group (*p* > .05), and the CNPase‐positive cells were the most in EAE 14 days, and there was significant difference in number of CNPase‐positive cells compared with EAE 1 day group (*p* < .05); the CNPase‐positive cells were decreased in 21 and 28 days group; the change trend at each time point in placebo treatment group was same with EAE group, and there was no significant difference between two groups (*p* > .05). In the MSCs treatment group, the CNPase‐positive cells were increased not obviously at 1 day, and increased significantly at 3 days, and reached peak from 7 to 14 days, then decreased, and there was significant difference in CNPase‐positive cells compared with the EAE group and the placebo treatment group (*p* < .05) (Table 4, Figure 6).

**TABLE 4:** Changes of CNPase‐positive cells in rat brain in each gorup (*n*/field, $\bar{X}$ ± *s*)

Groups	1 day	3 days	7 days	14 days	21 days	28 days
EAE	6.9 ± 3.8	7.3 ± 4.2	7.2 ± 4.3	13.6 ± 2.8^a^	14.0 ± 3.8^a^	13.8 ± 3.5^a^
Placebo treatment	6.3 ± 4.6	7.5 ± 3.8	6.8 ± 4.2	15.3 ± 3.5^a^	14.5 ± 3.2^a^	14.1 ± 2.8^a^
MSCs treatment	7.2 ± 2.8	15.3 ± 3.8^b^, ^c^	22.8 ± 3.6^b^, ^c^	30.6 ± 4.1^b^, ^c^	32.3 ± 4.4^b^, ^c^	28.9 ± 5.0^b^, ^c^

^a^ *p* < .05, compared with 1 day in the same group.

^b^ *p* < .05, compared between MSCs treatment group and EAE group.

^c^ *p* < .05, compared between MSCs treatment group and placebo treatment group.

## 4 DISCUSSION

In the central nervous system, the main function of OLG is the formation of myelin. In the EAE model with demyelination as the main pathological change, the affected cells are mostly OLG, so how to promote the recovery of OLG is the focus of attention. There are two general methods, one is to promote the regeneration of endogenous oligodendrocyte precursor cells (OPCs), and the other is the exogenous glial cell transplantation differentiate into functional OLG, so as to achieve the purpose of myelin regeneration.

**FIGURE 7:**
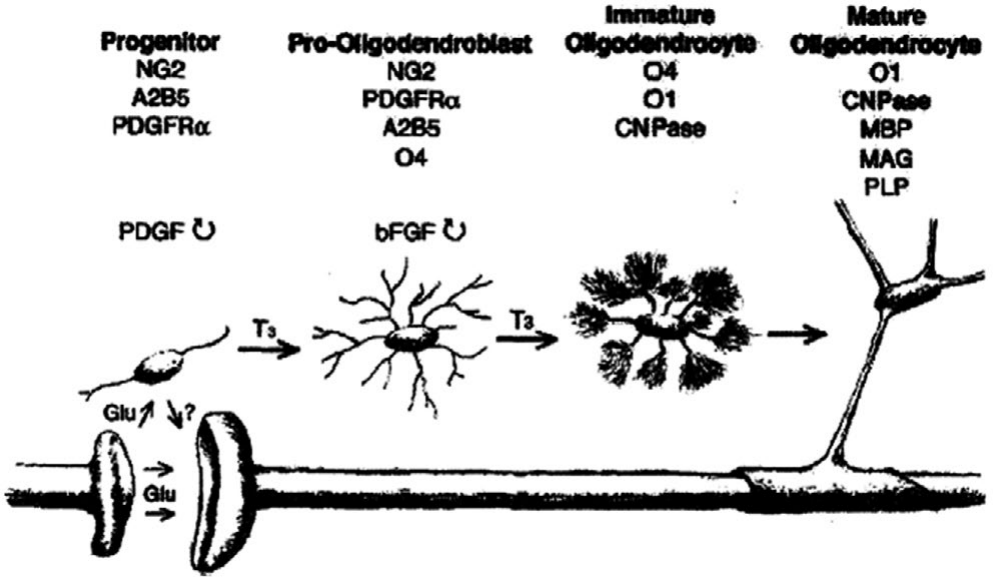
The specific antigens expressed at different developmental stages, and the corresponding antibodies have been generated

OLG originate in the neural epithelial cells of embryonic neural tube, and the morphology, expression products and functions of the OLG present a gradual and continuous process during the development from stem cells to mature OLG. Generally go through stem cells, OPCs, the former oligodendrocyte, immature and mature OLG, there are no strict boundaries of the stages, so the division of stages is relative. At present, the specific antigens expressed in different developmental stages have been identified and the corresponding antibodies have been generated (Deng & Poretz, 2002) (Figure 7), which makes it possible to further study OLG (Armstrong, 1998; Baumann & Dinh, 2001; Deng & Poretz, 2002). OLG, in addition to the formation of myelin sheaths for neuronal axonal segments, also secrete substances such as neurotrophic factors, growth factors, and axon growth inhibitory factors, together with other neuronal cells to regulate the microenvironment in the brain, and the satellite OLG around the cell bodies of neurons may have this function (Ludwin, 1997).

Our results showed that there are also OPCs in the brain of normal adult rats. The A2B5‐ and O4‐labeled oligodendrocyte progenitor cells and immature OLG are mainly distributed in the cerebral cortex and subcortical white matter. In addition, OPC also distributed in the corpus callosum, and in beaded arrangement; striatum, subventricular zone, and the cerebellum and other areas are distributed OPC (Blakemore & Keirstead, 1999; Roy et al., 1999). Studies have shown that rat OPC accounts for about 5%–8% of all nerve cells in the brain, and the proportion of OPC in human brain is still larger. Due to the different brain regions, the proportion of OPC is also different, OPC accounted 8%–9% of all nerve cells in the white matter region and 2%–3% in the cortical region, and the ratio of OPC to mature OLG in the cortex is approximately 1:1 (Chang et al., 2000; Dawson et al., 2003; Ohta et al., 2003). Although there is so much OPC in the adult rat brain, its specific role in the brain is unclear. In vitro studies have shown that OPC can affect the efficiency of synaptic transmission (Pfrieger & Barres, 1997), support neuronal survival (Sortwell et al., 2000), promote axonal growth (developmental phase) (Sanchez et al., 1996), and secrete growth factors to induce neuronal Na^+^ channel aggregation (Kaplan et al., 1997). In vivo studies show that OPC can form functional glutamate synapses with hippocampal CA1 neurons (Bergles et al., 2000). Some scholars believe that when the OLG in the brain are damaged and reduced, OPC can differentiate into mature OLG, thereby replenishing the damaged OLG and forming myelin sheaths for neuronal axons (Chari & Blakemore, 2002; Tanaka et al., 2003). It is also suggested that OPC in adult brain regulates the microenvironment, such as NG2, a chondroitin sulfate protein expressed on the cell membrane, can inhibit neuronal axon regeneration (Fawcett & Asher, 1999). There is also a view that OPC is probably the fifth nerve cell in the brain that is not known yet (the remaining four kinds are neurons, astrocytes, OLG, and microglia) (Greenwood & Butt, 2003).

The results of this study showed that there were more A2B5‐positive cells in the cerebral cortex of adult rats that are oligodendrocyte precursor cells. These cells have a small cell body and are round or oval. In the late EAE, the morphology of these cells changed, and the cell body became hypertrophic and heavily stained. Counting showed that the number of OPCs showed time‐history changes at different time points of EAE, the number of OPCs decreased at 1 day of EAE, and the decreasing was particularly significant at 3 and 7 days groups, then the number of OPCs began to increase, and reached to the largest number at 7 and 14 days of EAE, while the number of OPCs reduced significantly again at 28 days of EAE. These changes of OPCs at different time points of EAE suggested that it may be involved in the early pathogenesis and repair of EAE‐induced brain injury. Because OPC has only low migration capacity (Levine et al., 2001), the increase of OPCs should be the result of proliferation, not migrated from elsewhere, such as the subventricular zone of the lateral ventricle. OPC has highly sensitive to various types of nerve damage. The number of OPC was increased following cerebral ischemia, mechanical brain injury, inflammation, and seafine injection (Kondo & Raff, 2000). The mechanism of OPC proliferation increasing after brain injury is unclear. Literatures reported that some OPCs can express both neuronal markers (MAP2) and astrocyte markers (GFAP) (Kondo & Raff, 2000). Under in vitro regulation, OPCs can become a pluripotent stem cells, which not only can differentiate into mature OLG, but also can differentiate into astrocytes and neurons, therefore, it is reasonable to assume that OPC is the stem cell that helps damage tissue repair by providing myelinating cells (Ohta et al., 2003). The experimental results showed that mature brain has the endogenous protective mechanism of promoting myelin regeneration by replenishing OLG. Mature OPCs have the potential to differentiate into mature OLGs to repair damaged brain tissue, suggesting that OPC reactive hyperplasia can achieve the effect of tissue repair by compensating for OLG and remyelination after EAE.

In our experiment, we used O4 to mark immature OLG, an OLG surface marker, which mainly expressed on the cell membrane of late‐stage OPC and immature OLG. The results showed that the number of O4‐positive cells was decreased significantly at EAE1d, lasting from 7 to 14 days after EAE, suggesting that EAE induced significant immature OLG damage.

In myelin synthesis, OLG differentiation, hyperplasia occurred before myelin synthesis. During OLG differentiation and hyperplasia, OPC develops into immature OLG, while the latter differentiates into mature OLG. Mature OLG expresses CNPase, MBP and proteolipid protein (PLP) and so on. CNPase is a mature OLG marker mainly present in the evacuated myelin cell bodies. The results of this study showed that the expression of CNPase decreased significantly at EAE1d and remained at a lower level at 3 and 7 days. The decreasing of CNPase immunostaining suggested the injury of mature OLG. The CNPase expression gradually recovered at 14, 21, and 28 days.

In the placebo treatment group, the changes of A2B5, O4, and CNPase were the same as that of EAE group. There was no significant change in the morphology and number of A2B5, O4, and CNPase at 1 day after treatment in MSCs treatment group. The numbers of A2B5, O4, and CNPase increased significantly from 3 days after treatment and remained at a higher level at 7, 14, 21, and 28 days after treatment. These results indicated that the MSCs treatment can significantly increase the expression of OPCs, immature OLG, and mature OLG, thus laying a solid foundation for myelin regeneration.

## CONFLICT OF INTEREST

The authors declare that they have no competing interests.

## AUTHOR CONTRIBUTIONS

Zhang JM designed this study and analyzed the data; Wang H performed the experiment and analyzed the data; Fan YY and Yang FH wrote the manuscript. All authors read and approved the final manuscript.

## Data Availability

All data generated or analyzed during this study are included in this published article.
